# Versorgungsforschung in der Zahnmedizin in Deutschland

**DOI:** 10.1007/s00103-021-03356-3

**Published:** 2021-06-08

**Authors:** Fabian Huettig, Falk Schwendicke

**Affiliations:** 1 Poliklinik für Zahnärztliche Prothetik mit Propädeutik, Universitätsklinik für Zahn‑, Mund- und Kieferheilkunde, Universitätsklinikum Tübingen, Osianderstr. 2–8, 72076 Tübingen, Deutschland; 2 Abteilung für Orale Diagnostik, Digitale Zahnmedizin und Versorgungsforschung, Charité Universitätsmedizin Berlin, Aßmannshauser Straße 4–6, Berlin, 14197 Deutschland

**Keywords:** Orale Medizin, Mundgesundheit, Forschungsförderung, Ausbildung, Public Health, Oral medicine, Review, Education, Research funding, Public health

## Abstract

In den letzten 8 Jahren wurde in Deutschland nicht nur „mehr Versorgungsforschung in der Zahnmedizin“ gefordert, sondern auch geleistet. Insgesamt finden sich an 12 Medizinischen Fakultäten themenbezogene Aktivitäten der Zahn‑, Mund- und Kieferheilkunde in der Versorgungsforschung; deutschlandweit werden 9 Großprojekte verortet, die vom Bundesministerium für Bildung und Forschung oder vom Innovationsfonds gefördert werden. Gleichwohl ist der Bedarf an Versorgungsforschung größer als die jetzige Leistungsfähigkeit der universitären und außeruniversitären Zahnmedizin: Um eine nachhaltige, bedarfsgerechte und zukunftssichere zahnärztliche Versorgung aller Menschen in Deutschland gewährleisten zu können, bedarf es strukturierter, methodisch versierter und in die Versorgung hinein vernetzter Verbünde, die das wissenschaftliche Fundament für erwartete Versorgungsumbrüche legen können.

Der vorliegende Beitrag soll den Stand der Versorgungsforschung in der Zahn‑, Mund- und Kieferheilkunde in Deutschland beschreiben. Die wesentlichen Herausforderungen werden adressiert: Methodenkompetenz, Zugang zu Daten und deren Nutzung sowie die langfristige Perspektive dieses Forschungsbereichs. Derzeitige Forschungsaktivitäten und Infrastruktur inklusive Förder- und Fortbildungsinstrumente werden dargestellt.

Die Erkenntnisse aus der zahnärztlichen Versorgungsforschung in Deutschland können auch für andere Länder richtungsweisend sein; umgekehrt kann Versorgungsforschung Ansätze aus anderen Ländern sinnvoll in das deutsche Gesundheitssystem übertragen. Versorgungsforschende sollten sich professionalisieren und vernetzen. Nachhaltige Strukturen (Professuren, Mittelbau) und Rahmenbedingungen (Datennutzung, Förderung) sollten geschaffen und Forschungsergebnisse zeitnah verwertet werden.

## Abstract (en)

Over the past eight years, the continuous call for “more oral health services research” in Germany has been (at least partially) answered. Today, relevant oral and dental health services research activities can be found at 12 medical faculties in Germany and nine large-scale projects are funded by the German Ministry for Education and Research (BMBF) or the Innovation Fund of the Federal Joint Committee (G-BA). However, the demand for high-level research in this field exceeds the current capacities of university and non-university dentistry; structured and methodically competent research networks that are cross-linked within the dental and public health services are required. These would allow research to assure sustainable and demand-oriented dental services, scientifically supporting the demographically, economically, and socially driven major changes to be expected in the coming years.

The article describes the current status of dental and oral health services research in Germany and addresses major challenges: methodological competence, data accessibility and usage, and the prospects for this field of research. Therefore, it takes stock of existing research activities and infrastructure (including educational and funding opportunities).

Knowledge obtained from dental and oral health services research in Germany can be pioneering for other countries, while researchers in Germany may be able to adopt and adapt servicing strategies from other countries and health systems for Germany. To achieve these aims, dental and oral health services researchers should professionalize and network. Sustainable structures (professorships, staff) and frameworks (data availability, funding) should be established and findings promptly translated into use.

## Einführung

Dass Versorgungsforschung in der Zahnmedizin ein Schwerpunkt der zukünftigen Forschung sein muss, wird seit über 10 Jahren verstärkt proklamiert [1–4]. Eine „Initialzündung“ zur Dynamisierung der zahnmedizinischen Versorgungsforschung in Deutschland gab im Jahr 2012 die gemeinsame Tagung der Deutschen Gesellschaft für Zahn‑, Mund- und Kieferheilkunde (DGZMK), des Deutschen Verbands für Gesundheitswissenschaften und Public Health (DVGPH) und des Deutschen Netzwerks für Versorgungsforschung (DNVF). Allerdings liegt die deutsche Versorgungsforschung an sich, und besonders auch die im Bereich Zahnmedizin, immer noch weit hinter den Entwicklungen in anderen Ländern Europas zurück.

**Figure 1:**
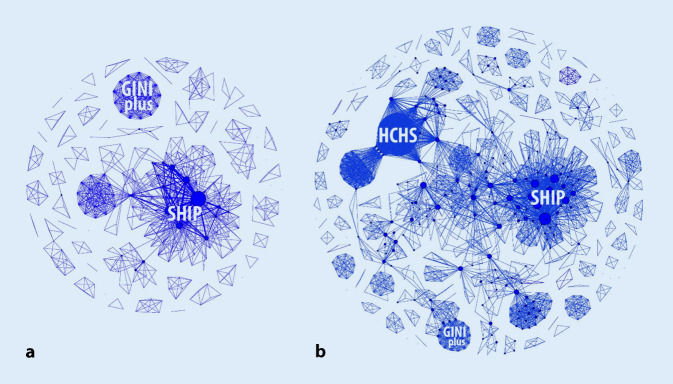

Eine Analyse von PubMed-gelisteten Publikationen zur zahnärztlichen Versorgungsforschung in bzw. über Deutschland^1^ von 1997 bis Ende 2020 zeigt eine zunehmende Vernetzung von Forschungsgruppen sowie eine Schwerpunktbildung von Publikationen aus wenigen Großprojekten wie etwa der Study of Health in Pomerania (SHIP) oder der Hamburg City Health Studie (HCHS; Abb. 1). Wenige maßgebliche Player, oft gemeinsam in größeren Verbundstrukturen, tragen den großen Teil der Publikationen. Die zeitliche Analyse des Netzwerks und der Publikationsaffiliationen zeigt zudem, dass zahnmedizinische Versorgungsforschung auch zunehmend durch Zahnmediziner (und nicht z. B. aus der allgemeinmedizinischen/soziologischen Versorgungsforschung heraus) betrieben wird.

Der vorliegende Artikel soll den Stand der Versorgungsforschung in der Zahn‑, Mund- und Kieferheilkunde sowie die Voraussetzungen für deren erfolgreiche zukünftige Entwicklung beschreiben. Dazu adressiert er die wesentlichen Herausforderungen (Methodenkompetenz, Zugang und Nutzung von vorhandenen Daten, langfristige Perspektive dieser Forschungsebene in der Zahnmedizin), die vorhandene Infrastruktur (Forschende, Institutionen) inklusive Förder- und Fortbildungsinstrumente sowie derzeitige Forschungsaktivitäten. Daraus abgeleitet wird, wie diese Ressourcen perspektivisch und themenbezogen für eine effiziente zahnärztliche Versorgungsforschung vernetzt werden können und wo Bedarf an politischen, juristischen und/oder letztlich finanziellen Maßnahmen besteht.

## Versorgungsforschende in der Zahn‑, Mund- und Kieferheilkunde

Der folgende Überblick identifiziert derzeitige und potenziell Forschende im Bereich der zahnärztlichen Versorgungsforschung, die zu einer höheren Vernetzung der Forschungsaktivitäten und Bearbeitung von zukünftigen Themen beitragen können.

### Einrichtungen der zahnärztlichen Selbstverwaltung

Die Selbstverwaltung der Zahnärzteschaft hat für die Berufsausübung und Leistungserbringung mit den Zahnärztekammern (Bundes- und Landeszahnärztekammern – BZÄK/LZÄK) wie auch den kassenzahnärztlichen Vereinigungen auf Landes- und Bundesebene (Kassenzahnärztliche Bundesvereinigung – KZBV und kassenzahnärztliche Vereinigungen – KZVen) 2 Strukturen, die Versorgungsforschung direkt oder indirekt betreiben oder befördern. Deren Institutionen stellen wichtige Daten aus der zahnärztlichen Versorgung zur Verfügung und bereiten diese regelmäßig auf. So sind die beiden statistischen Jahrbücher (der BZÄK und der KZBV) zur zahnärztlichen Versorgung eine maßgebliche Ergänzung der (Gesundheits‑)Statistik der Länder und des Bundes. Die KZBV erhebt etwa zusätzlich mit dem „Zahnärzte-Praxis-Panel“ (ZäPP) Daten zur Kosten- und Versorgungsstruktur der vertragszahnärztlichen Praxen. Die BZÄK veröffentlichte 2020 zum dritten Mal den Qualitätsreport.

### Das Institut der Deutschen Zahnärzte in Köln

Gemeinschaftlich via BZÄK und KZBV finanziert die deutsche Zahnärzteschaft selbstständig und nachhaltig seit 1987 das Institut der Deutschen Zahnärzte (IDZ) gemeinsam mit dem als Stabsstelle zugeordneten Zentrum Zahnärztliche Qualität (ZZQ). Themenfelder, die das IDZ abbildet, sind die Gesundheitsversorgungsforschung und -epidemiologie, Gesundheitsökonomie und -systemforschung, zahnärztliche Professionsforschung, Medizinsoziologie und Gesundheitspsychologie [5]. Das IDZ ist damit entfernt vergleichbar mit dem „Zentralinstitut für die kassenärztliche Versorgung“ (welches als Stiftung der Kassenärztlichen Bundesvereinigung finanziert wird). Federführend begleitet das IDZ die Deutschen Mundgesundheitsstudien (DMS) – derzeit in Vorbereitung der 6. Durchführung. Das IDZ stellt seine Arbeit und Ergebnisse in Form von Publikationen frei zur Verfügung (Open Access) und bietet zudem gebundene Monografien zum Kauf an.

### Fortbildungseinrichtungen der Kammern

Zahnärztekammern betreiben darüber hinaus auf Landesebene Fortbildungseinrichtungen für ihre Mitglieder. Davon sind 2 in Baden-Württemberg selbst in der Patientenversorgung tätig: Das Zahnmedizinische Fortbildungszentrum (ZFZ, Stuttgart) und die Akademie für Zahnärztliche Fortbildung Karlsruhe. Beide sind durch ihr Praxisnetzwerk auch in der Versorgungsforschung aktiv in Hochschulprojekte (siehe unten) eingebunden: die Karlsruher Akademie etwa zur Frage endodontischer Therapieoutcomes [6] und im Horizont 2020 EU-Projekt ADVOCATE [7] sowie im Innovationsfondsprojekt Dent@Prevent, das ZFZ etwa zur Periimplantitisprävention [8].

### Öffentliches Gesundheitswesen und die DAJ

Die Deutsche Arbeitsgemeinschaft für Jugendzahnpflege (DAJ) ist die bundesweite Institution öffentlicher zahnmedizinischer Gesundheitsfürsorge in Deutschland. Leistungen der DAJ werden im vorliegenden Themenheft beschrieben (siehe Beitrag von Schmoeckel et al.). Die zahnärztlichen Dienste im Öffentlichen Gesundheitsdienst (ÖGD) sind, je nach Bundesland verschieden, auf Ebene der Kommunen oder Landkreise in den Gesundheitsämtern organisiert. Ihnen kommen Aufgaben der Beratung und Aufklärung der Bevölkerung zu allen Fragen der Zahnmedizin sowie Schulung von Multiplikatoren, umfangreiche Maßnahmen zur Prävention (u. a. Gruppenprophylaxe), die infektionshygienische Überwachung von zahnärztlichen Einrichtungen sowie ein erheblicher Anteil der Bewertung des Gesundheitszustandes der Bevölkerung zu (Bundesgesundheitsberichterstattung). Für die zahnärztlichen Daten sollten diese Datenerhebungen flächendeckend einheitlich nach Maßgaben der Weltgesundheitsorganisation (WHO) der Basic Methods for Oral Health Surveys von 2013 erfolgen, dies ist bisher jedoch nicht einheitlich gegeben. Die Notwendigkeit zur Weiterentwicklung des ÖGD ist im Jahr 2013 im Positionspapier des Landkreistages sowie 2016 nach Verabschiedung des Präventionsgesetzes angemahnt worden [9]. Der Kontrast zwischen den begrenzten Leistungsmöglichkeiten des ÖGD und dem hohen Bedarf an öffentlicher (zahnärztlicher) Gesundheitsfürsorge ist besonders deutlich in der Coronapandemie zutage getreten.

### Bundeswehr

Die Bundeswehr bietet in 140 Zahnarztgruppen freie Heilfürsorge an und unterhält wissenschaftliche Institute. Das Kommando Sanitätsdienst verwaltet longitudinale Daten der Grund- und fach(zahn)ärztlichen Versorgung und führt die „Planung und Steuerung der zahnärztlichen Versorgung der Bundeswehr“ u. a. mit Entwicklung des One-Health-Ansatzes bei der Versorgung der Soldatinnen und Soldaten; das bedeutet die feste Einbindung der Zahnmedizin in die Heilfürsorge zur Allgemeingesundheit [10]. Dies eröffnet Kooperationsmöglichkeiten, Erkenntnisse zu generieren sowie selbige in die zivile Versorgung und Versorgungsplanung zu transferieren.

### Krankenversicherungen

Die Krankenversicherungen besitzen teilweise wissenschaftliche Institute, die ebenfalls Beiträge zur zahnärztlichen Versorgungsforschung leisten oder leisten könnten. So publiziert das Institut für Gesundheitssystemforschung der Barmer im Bereich Zahnmedizin mit universitärer (Dresden) und externer Unterstützung jährlich den „Barmer Zahnreport“. Die AOK betreibt seit 1970 das Forschungs- und Beratungsinstitut des AOK-Systems (WidO). Die Techniker Krankenkasse hat ihr wissenschaftliches Institut WINEG aufgelöst und die Versorgungsforschung in die jeweiligen Fachabteilungen dezentralisiert. Die privaten Krankenversicherungen (PKVen) unterhalten in Köln das Wissenschaftliche Institut der PKVen (WIP).

### Die NAKO Gesundheitsstudie

Der eingetragene Verein NAKO führt unter Finanzierung des BMBF, der Länder und der Helmholtz-Gemeinschaft an 18 Studienzentren eine auf 20 bis 30 Jahre angesetzte Langzeitbeobachtung zum medizinischen und sozialen Status von 200.000 Personen zwischen 20 und 69 Jahren in Deutschland durch. Auch eine zahnmedizinische Untersuchung ist seit Beginn der Vorbereitungen im Jahr 2012 Teil der NAKO Gesundheitsstudie [11, 12]. Erhoben werden hierbei auf 2 Erhebungslevels mittels Befragung und Untersuchung von Teilnehmenden neben einer Speichelprobe ein Basisdatensatz zu Parodontitis, zur Funktion des Kiefers (Gelenk und Muskulatur) und der Verzahnung, zur mundgesundheitsbezogenen Lebensqualität (OHIP-5) sowie zur Anzahl der Zähne und dem Vorliegen von herausnehmbarem Zahnersatz. An der NAKO sind 10 zahnärztliche Lehrstühle beteiligt: Mund‑, Kiefer- und Gesichtschirurgie (1), Kieferorthopädie (1), Zahnerhaltung (5), Prothetik (3) an insgesamt 9 Standorten (Kiel, Greifswald, Hamburg, Münster, München, Heidelberg, Tübingen, Würzburg, Homburg/Saar).

### Versorgungsforschung an Lehrstühlen für Zahn‑, Mund- und Kieferheilkunde

Mittlerweile findet ein großer Anteil der zahnärztlichen Versorgungsforschung, teils in Zusammenarbeit mit den zuvor genannten Akteuren, an Lehrstühlen der Zahn‑, Mund- und Kieferheilkunde statt. Zunächst sollen die klassischen zahnmedizinischen Lehrstühle vorgestellt werden, dann die mittlerweile eigenständigen Lehrstühle, die schwerpunktmäßig u. a. Versorgungsforschung betreiben.

Durch ihre Integration in die SHIP-Studie sind die Lehrstühle für Parodontologie, Zahnerhaltung und Prothetik in Greifswald hochaktiv in der universitären Versorgungsforschung [13]. Auch der Lehrstuhl für Zahnärztliche Prothetik der TU Dresden ist seit 2015 am jährlichen, mit Schwerpunktthemen publizierten Zahnreport der Barmer in der Versorgungsforschung sichtbar. An der Universität Hamburg ist der Lehrstuhl für Zahnärztliche Prothetik erfolgreich in der HCHS engagiert, u. a. zu Fragen der mundgesundheitsbezogenen Lebensqualität und Patientenautonomie [14, 15]. Die Polikliniken für Zahnerhaltung an den Universitätskliniken Freiburg, Göttingen (gemeinsam mit der Zahnersatzkunde) und Leipzig haben praxisbasierte Forschungsnetzwerke geschaffen [8]; letzterer Verbund widmet sich schwerpunktmäßig den Zusammenhängen von Zahnmedizin und chronischen Allgemeinerkrankungen [16, 17]. Weiter finden sich Aktivitäten an der Universität Münster – etwa zum Register Oraler Manifestationen Seltener Erkrankungen [18] – und der Universitätszahnklinik Witten/Herdecke, u. a. zu den Themen „Zuckerkonsum“ und „Menschen mit Behinderung“ [19].

### Professuren für zahnärztliche Versorgungsforschung

Analog zur Public-Health-Initiative der 1990er-Jahre fordert das DNVF bereits seit Längerem den Ausbau einer flächendeckenden wissenschaftlichen „Grundausstattung“ für Versorgungsforschung an den Hochschulen [20, 21]. Außerhalb der Zahnmedizin sind heute über 20 universitäre Versorgungsforschungseinrichtungen etabliert. Forschende der Zahn‑, Mund- und Kieferheilkunde finden zwar teilweise Anschluss an diese Versorgungsforschungseinrichtungen, allerdings fehlt es für die Zahnmedizin noch an „Inkubatoren“ für Studierende und (Nachwuchs‑)Wissenschaftler des zahnärztlichen Fächerspektrums. So sind derzeit nur 3 Professuren für die Zahnmedizin erfolgreich etabliert worden.

*Christian-Albrechts-Universität zu Kiel:* Im März 2014 wurde die erste Professur für Versorgungsforschung in der Zahnmedizin „Prävention und Versorgung in der Zahnheilkunde“ eingerichtet und Frau Prof. Dr. med. dent. Katrin Hertrampf, MPH, berufen.

*Charité – Universitätsmedizin Berlin: *Im Jahr 2020 richtete die Charité – Universitätsmedizin einen Lehrstuhl für „Orale Diagnostik, Digitale Zahnheilkunde und Versorgungsforschung“ mit einer eigenständigen zugeordneten klinischen Abteilung ein.

*Westfälische Wilhelms-Universität Münster:* Im Juli 2019 wurde die W3-Professur „Versorgungsforschung in der Zahnmedizin“ am Zentrum für Zahn‑, Mund- und Kieferheilkunde Münster ausgeschrieben und zum 01.02.2021 besetzt.

*Ruprecht Karls Universität Heidelberg:* Im Jahr 2018 wurde die W3-Professur für „Translationale Gesundheitsökonomie in der Zahnmedizin“ ausgeschrieben. Zum Zeitpunkt der Drucklegung ist diese Professur noch nicht besetzt.

## Zahnmedizinstudium und Kompetenzerwerb in zahnärztlicher Versorgungsforschung

Zahnärztliche Versorgungsforschung, Epidemiologie, Public Health und Versorgungsforschung sind in der noch bis Oktober 2021 gültigen Version der Approbationsordnung für Zahnärzte (AOZ) weder als Fach noch inhaltlich strukturiert verankert. Da es sich bei allen 3 Teilbereichen um nicht originär „zahnärztliche“, sondern vielmehr medizinsoziologische/mathematische/sozialwissenschaftliche Fachgebiete handelt, ist eine Ausprägung an den Medizinischen Fakultäten – sofern diese hier nicht einen gesonderten Schwerpunkt gesetzt haben (s. oben) – entsprechend gering. Allerdings besteht erheblicher Bedarf, alle 3 Teilbereiche in einem zeitgemäßen Studium der Zahnmedizin abzubilden. In der neuen AOZ wird dies in 2 Querschnittsbereichen (QB) mit insgesamt ca. 20 Zeitstunden erfolgen. Somit entspricht es maximal einer Einführungsveranstaltung. Auch der im Jahr 2015 konsentierte Nationale Kompetenz-basierte Lernzielkatalog Zahnmedizin (NKLZ) hat nur in 37 von 1408 Lernzielen einen Bezug zur Versorgungsforschung.

Daneben existieren Weiterbildungsangebote ohne strukturierten Charakter. Maßgeblich zu nennen sind die Spring School des DNVF wie auch Methodenworkshops der Arbeitsgruppe „Erhebung und Nutzung von Sekundärdaten“ (AGENS). Über bundesweite Fort- und Weiterbildungsveranstaltungen – aber auch aktuelle Förderformate – informiert zuverlässig seit einigen Jahren die Koordinierungsstelle Versorgungsforschung der Albert-Ludwigs-Universität Freiburg [22].

So bleibt festzustellen, dass es einen Bedarf für ein strukturiertes Qualifizierungsprogramm gibt, welches Nachwuchswissenschaftler berufsbegleitend in den Grundlagen ausbildet und weitere Vertiefungsmöglichkeiten anbietet.

## Förderformate für zahnärztliche Versorgungsforschung

Derzeit bestehen keine speziellen Förderformate für die Zahn‑, Mund- und Kieferheilkunde. Die klassischen zahnmedizinischen Fächer befinden sich daher mit „ihren“ Themen nicht nur in Konkurrenz zueinander, sondern auch mit den „etablierten“ Disziplinen in der Gesundheitssystem- und Versorgungsforschung. Deshalb ist es für die Zahnmedizin unumgänglich, Verbundprojekte aufzusetzen; nur so lassen sich auch die gestiegenen methodischen Anforderungen erfüllen. Dies setzt, wie eingangs dargestellt, die Vernetzung von Forschenden voraus, sowohl national als auch international. Hierfür sind Fördermittel bei einer Reihe von Fördergebern beantragbar:

### EU-Förderung

Im Rahmen des europäischen Forschungsrahmenprogramms Horizont 2020 legte die EU-Kommission mehrere thematische Schwerpunkte mit Bezug zur Versorgungsforschung. In der Förderdatenbank CORDIS findet sich das 2015–2019 mit 6 Mio. EUR geförderte zahnmedizinische Versorgungsforschungsprojekt „Added Value for Oral Care“ (ADVOCATE; #635183). Einer der 3 europäischen Initiatoren (Prof. Dr. Dr. Listl) koordinierte am Universitätsklinikum Heidelberg gesundheitsökonomische Analysen, die Auswertung von Routinedaten aus 6 europäischen Ländern sowie die Konsentierung von Indikatoren zur zahnmedizinischen Versorgungsqualität. Daneben konnte ein interaktives Dashboardsystem zur Förderung der Versorgungsqualität erfolgreich erprobt werden.

### DFG-Förderung

Die Deutsche Forschungsgemeinschaft (DFG) hat bereits im Jahr 2010 eine Stellungnahme „Versorgungsforschung in Deutschland: Stand – Perspektiven – Förderung“ veröffentlicht. Maßgeblich wurden seither 8 Nachwuchsakademien für Versorgungsforschung gefördert. In der Förderdatenbank „gepris.de“ finden sich neben 3 Nachwuchsakademien Zahnmedizin noch ein Projekt an der Charité – Universitätsmedizin Berlin (#445925495, seit 2020) sowie ein Projekt am Universitätsklinikum Hamburg-Eppendorf (UKE; #232689885, 2012–2014), die im weitesten Sinne der Versorgungsforschung Zahnmedizin zuzurechnen sind. Da es sich bei Versorgungsforschung um den Grenzbereich von Wissenschaft und Gesundheitsversorgung handelt, sind auch die Mittel und Antragsthemen der DFG für diesen Bereich begrenzt.

### BMBF-Förderung

Das Bundesministerium für Bildung und Forschung (BMBF) hat 2013 den „Aktionsplan Versorgungsforschung“ veröffentlicht und bis 2016 bereits über 50 Mrd. EUR verausgabt. Bis dato finden sich auch 2 aus der Zahnmedizin stammende Projekte, die durch das BMBF gefördert werden:

Die abteilungsübergreifende Nachwuchsforschergruppe an der Charité – Universitätsmedizin Berlin bearbeitet das Projekt „TAILOHR – Entwicklung, Adaptation und Implementierung einer Intervention zur Verbesserung der Mundgesundheit in der stationären Altenpflege“.

Das Zentrum für Experimentelle Medizin, Institut für Medizinische Biometrie und Epidemiologie am Universitätsklinikum Hamburg-Eppendorf untersucht in einem Teilprojekt von „PERGOLA2“, wie Aussiedler im Vergleich zur deutschen Gesamtbevölkerung Vorsorgeuntersuchungen sowie medizinische und zahnmedizinische Leistungen in Anspruch nehmen.

Nach einer ersten Phase im Jahr 2020 bewilligte der Bundestag Haushaltsmittel für den Ausbau des Netzwerks Universitätsmedizin (NUM) als innovatives Förderformat. Das BMBF „fördert mit 150 Mio. Euro den Aufbau des Forschungsnetzwerks … zur Bewältigung der Covid-19 Pandemie“ (https://www.netzwerk-universitaetsmedizin.de/). Dabei liegt ein Schwerpunkt bei der „Versorgungsforschung, deren Ergebnisse gemäß dem translationalen Ansatz direkt in das Versorgungsgeschehen bzw. Krisenmanagement einfließen bzw. es unterstützen sollen. Zudem sollen nach Möglichkeit nachhaltige Strukturen etabliert werden, die auch über das Projekt hinaus Wirksamkeit in der zukünftigen Zusammenarbeit entfalten.“ (https://www.netzwerk-universitaetsmedizin.de/)

### Förderung durch den Innovationsfond des Gemeinsamen Bundesausschusses (G-BA)

Der G‑BA förderte im Rahmen des Innovationsfonds bisher 7 Projekte mit zahnmedizinischem Fokus:

*Dent@Prevent*: In der ersten Runde des Innovationsfonds wurde an der Sektion „Translationale Gesundheitsökonomie in der Zahnmedizin“ vom Universitätsklinikum Heidelberg das Projekt Dent@Prevent gefördert [23]. Es „will einen methodischen Beitrag für eine verbesserte Qualität und Ressourcen-Allokation in der Versorgung von Patienten mit zahnmedizinischen und chronischen Erkrankungen leisten“ [24].

*MundZaRR* – Mundgesundheitsverbesserung durch zahnärztlich delegierte, pflegebegleitende Remotivation und Reinstruktion: Das von Frau Prof. Dr. Hertrampf (Universität Kiel) geleitete Verbundprojekt (Köln, Jena, Charité – Universitätsmedizin Berlin) zielt darauf ab, das Konzept für Alter und Behinderung (AuB-Konzept) an die Anforderungen in stationären Pflegeeinrichtungen anzupassen. Langfristig sollen dadurch die Mundgesundheit und damit auch die Lebensqualität der Bewohner in der stationären Seniorenpflege verbessert werden [25].

*InSEMaP* – Interaktionen von Systemischen Erkrankungen und Mundgesundheit bei ambulanter Pflegebedürftigkeit: Unter Leitung des Instituts für Gesundheitsökonomie und Versorgungsforschung an der Universität Hamburg zielt das Projekt auf „die Verbesserung der zahnärztlichen Versorgung von ambulant gepflegten Menschen vor dem Hintergrund einer oftmals stark abnehmenden Inanspruchnahme zahnärztlicher Leistungen nach erfolgtem Pflegebeginn“ ab.

*MundPflege* – Mundgesundheit bei Pflegebedürftigen: Das an der Universität Bremen geführte Projekt untersucht, wie die „Mundgesundheit ambulant versorgter pflegebedürftiger Personen erhalten und verbessert werden kann. Hierzu werden niedrigschwellige und präventive zahnmedizinische Leistungen im eigenen Wohnumfeld angeboten. Die an dem Projekt teilnehmenden Pflegebedürftigen und ihre Pflegepersonen erhalten zudem eine individualisierte Schulung zur Verbesserung der Mund- und Prothesenpflege“.

*MuMi* – Förderung der Mundgesundheitskompetenz und Mundgesundheit von Menschen mit Migrationshintergrund: Das am Lehrstuhl für Zahnärztliche Prothetik der Universität Hamburg von Frau PD Dr. Aarabi geleitete Projekt „MuMi“ untersucht, ob eine App die Mundgesundheit von Patientinnen und Patienten mit Migrationshintergrund verbessert. Dafür werden 800 Patienten mit Migrationshintergrund und 200 Patienten ohne Migrationshintergrund, die mittels dieser App geschult werden, verglichen mit einer gleichen Anzahl an Patienten, die diese App nicht nutzen.

*EFAFU* – Effekte von Gebissanomalien auf Mundgesundheit und -funktion: Die Abteilung für Kieferorthopädie an der Universität Greifswald leitet das von allen zahnärztlichen Fachdisziplinen getragene Projekt zur Kurz- und Langzeitabschätzung der Effekte von Gebissanomalien und kieferorthopädischen Behandlungen auf die Mundgesundheit.

*IpKiSuN* – Unterstützende Intensivprophylaxe für Kinder mit zahnärztlicher Sanierung unter Narkose: Ebenfalls an der Universität Greifswald und geleitet durch die Abteilung Präventive Zahnmedizin und Kinderzahnheilkunde erforscht das Projekt, ob „die Mundgesundheit [durch] schon bestehende Vorbeugungsleistungen nachhaltig“ bei betroffenen Kindern verbessert wird. Gleichzeitig wird berechnet, ob sich die Maßnahmen auch in geringeren Behandlungskosten niederschlagen.

### Förderung durch das Bundesland Baden-Württemberg

Das Land Baden-Württemberg hat – bisher einzigartig – an 3 universitären Standorten Koordinierungszentren für Versorgungsforschung finanziert und zusätzlich im Rahmen von 3 Nachwuchsakademien mit 1,2 Mio. EUR zwischen 2011 und 2017 30 Projekte gefördert, darunter 3 zahnmedizinische an der Karlsruher Akademie [6], am Universitätsklinikum Tübingen [26] sowie am Lehrstuhl Soziologie der Universität Konstanz die „Pilotstudie zur zahnärztlichen Betreuung von Pflegeeinrichtungen nach Einführung von Kooperationsverträgen gemäß §119b SGB V“.

### Förderung im internationalen Rahmen

Durch erfolgreiche Kooperation von Prof. Dr. Dr. Listl (Universitätsklinikum Heidelberg) mit der University of Maryland (USA) wurde in dem Projekt „Dental Coverage Transitions, Utilization and Retirement“ (#3R01DE021678-06S1) durch die US-amerikanischen National Institutes of Health (NIH) erstmals ein zahnmedizinisches Versorgungsforschungsprojekt mit deutscher Beteiligung gefördert. Es untersuchte die zahnärztliche Versorgung von Personen, die das 50. Lebensjahr überschritten hatten und entweder in den USA oder europäischen Ländern (einschließlich Deutschland) lebten.

## Themenfelder für die zahnärztliche Versorgungsforschung bis 2040

In der im Januar 2021 durch den WHO-Exekutivrat verabschiedeten Resolution zur Mundgesundheit sowie in dem zeitgleich durch den Weltzahnärzteverband (FDI) veröffentlichten Strategiepapier „FDI Vision 2030“ wird eine Reihe von Themen angemahnt, die auch für die deutsche Versorgungsforschung relevant sind ([27, 28]; siehe Infobox). Besonders hervorzuheben ist die nachhaltige Verankerung der zahnmedizinischen Versorgung in der universellen Gesundheitsversorgung (UHC), für die Integration von zahnmedizinischer und medizinischer Versorgung, für die Stärkung von Public-Health-Prävention sowie für eine verbesserte Ressourcenplanung und Qualitätsförderung – dies schließt die Relevanz der Versorgungsforschung mit Professionalisierung in universitärer Forschung und Lehre ein.

Ein Teil dieser Aspekte soll – mit Rücksicht auf die Bestandsaufnahme – hier detaillierter aufgegriffen werden. Die WHO-Resolution zur Mundgesundheit und der FDI-Report „Vision 2030“ verweisen übrigens auch auf Ergebnisse zahnmedizinischer Versorgungsforschung aus Deutschland. Dabei insbesondere auf ökonomische Implikationen von Zahnerkrankungen sowie die Zusammenhänge zwischen chronisch-systemischen Erkrankungen und Zahnerkrankungen [29, 30].

### Versorgungsbedarfe, Versorgungsgerechtigkeit, Zugang zu Versorgung

Versorgungsbedarfe bis zum Jahr 2030 sind bereits umrissen worden [31–34] – und somit zwingend auch die Konsequenz, diese im Versorgungssystem mit einer kontinuierlichen Veränderung in der Patientenstruktur zu berücksichtigen. Neben den in diesem Themenheft dargestellten Herausforderungen für Menschen mit Behinderung und höheren Alters (siehe Beiträge Schulte und Schmidt bzw. Nitschke und Hahnel) betrifft Versorgungszugang und -gerechtigkeit auch zunehmend Migranten und Personen/Familien mit Migrationshintergrund und die damit verbundenen sprachlichen wie auch ethnischen und religiösen Herausforderungen für die Versorgungsstrukturen [35]. Auch wird der Zugang zu zahnärztlicher Versorgung im ländlichen Raum ein zentrales Thema sein.

### Mundgesundheit als Teil der Allgemeingesundheit: Universal Health Coverage

Die zahnärztliche Unterstützung zur Prävention und Therapie chronischer Volkskrankheiten, z. B. neurodegenerative Erkrankungen, Diabetes, Herz-Kreislauf-Erkrankungen und deren Risikofaktoren (Rauchen, Zuckerkonsum, Adipositas), die Schlafmedizin (Apnoetherapie) oder die Prävention von Missbrauch im Kindes- und Jugendalter ist durch den gemeinsamen Risikofaktorenansatz ein Schlüssel für „mehr Zahnmedizin“ in der Medizin.

Dies bedarf der Integration der Zahn‑, Mund- und Kieferheilkunde in der Universal Health Coverage (dt.: allgemeine Gesundheitsabsicherung) durch interdisziplinäre Kooperation zwischen Zahn‑, Allgemein- und Fachärzten sowie den einschlägigen Heilberufen, insb. Altenpflege, Physiotherapie, Logopädie. Dieser Komplex betrifft die (gesetzlichen, ökonomischen, soziologischen) Versorgungs- und Organisationsstrukturen und ihre Akteure (einschließlich Studierende/Auszubildende).

Auch sollten die Auswirkungen zahnmedizinischer Erkrankungen und Therapien auf die Lebensqualität in allen Altersphasen evaluiert werden, inklusive der Aufwände für das Gesundheitssysteme bzw. den Patienten (Gesundheitsökonomie) und der Auswirkungen, Barrieren und fördernden Faktoren zur Umsetzung von Gesundheitsmaßnahmen in verschiedenen Settings (Praxis, Klinik, Pflegeheim; [36]).

### Versorgungsstrukturen

Die Versorgungstrukturen werden zwar wesentlich politisch gesteuert, folgen allerdings auch ökonomischen und soziologischen Voraussetzungen, wie etwa bürokratischer Belastung, unternehmerischen Risiken oder weitergehenden und einem Wandel unterliegenden Lebensentwürfen [37, 38]. Neben solchen Einflüssen auf ärztliche Leistungserbringer kann/wird es – etwa durch EU-Gesetzgebung wie auch technologischen Wandel – zu einer Veränderung von Berufsbildern kommen: z. B. die Akademisierung von Assistenzberufen und Veränderungen in der Delegation derzeit ärztlicher Obliegenheiten. Technologisierung und Medizinprodukteregulation verändern das Handwerk der Zahntechnik (s. „Integration neuer Technologien“).

Neben etablierten Versorgungsstrukturen ist der Stellenwert der öffentlichen Gesundheitsfürsorge rund um die Zahn‑, Mund- und Kieferkrankheiten zu berücksichtigen und neu zu bestimmen, u. a. um den Herausforderungen benachteiligter Gruppen von Menschen zu begegnen.

### Epidemiologie der Zahn‑, Mund- und Kieferkrankheiten/Mundgesundheit

Die Stärkung des öffentlichen zahnmedizinischen Gesundheitsdienstes ist auch zur besseren Überwachung der Mundgesundheit in der Bevölkerung sinnvoll. Dadurch wäre neben den genannten Präventionsaspekten auch die kontinuierliche Bereitstellung von Gesundheitsdaten möglich, bei entsprechender Ausstattung (s. „Integration neuer Technologien“) und standardisierter, kompatibler Datenerhebung/-haltung/-teilung.

### Nachhaltigkeit

Die zahnärztliche Versorgung sowie die (häusliche) Prävention von Zahn‑, Mund- und Kieferkrankheiten sind bisher unzureichend hinsichtlich der Nachhaltigkeit wissenschaftlich beurteilt worden. Dies betrifft die Versorgung mit Amalgam [39], aber auch die Umweltbelastung durch Mikroplastik aus Mundhygieneartikeln, die steigende Anzahl von Einmalartikeln in der Versorgung wie auch Energie- und Chemikalieneinsatz und Feinstaubfreisetzung bei der Herstellung (z. B. Rechenleistung, 3‑D-Druck, CAM-Schleifen) und Aufbereitung (Desinfektion, Sterilisation) von Medizinprodukten.

### Integration neuer Technologien

Neue Technologien, z. B. digitale Gesundheits- und Medizinanwendungen (Apps) sowie deren Daten, können ebenso wie die massiven Datenmengen aus digitalen Routinedaten (Texte, Laborwerte, Bildgebung) und Sekundärdaten (Abrechnungsdaten) aus der Krankenversorgung für Prävention, Diagnostik und Therapie eingesetzt werden. Dazu kommen neue Instrumente, u. a. aus dem Feld der künstlichen Intelligenz (KI), Decision Support Systems, aber auch die Telezahnmedizin den Heilberufen unterstützend zu Hilfe. Fragen zur Robustheit und zum Nutzen, zur Evidenzbasierung und Sicherheit, zu Datenschutz und dem Einfluss auf bestehende Versorgungsprozesse und -strukturen sowie der Einsatz für Versorgungsforschung und -steuerung (s. „Versorgungsbedarfe, Versorgungsgerechtigkeit, Zugang zu Versorgung sowie Versorgungsstrukturen“) sind zu klären. Der neu gegründete Arbeitskreis KI in der Zahnmedizin AK AIDM (Artificial Intelligence in Dental Medicine) der DGZMK sowie die Arbeitsgruppe Zahnmedizinische Diagnostik und Digitale Zahnheilkunde in der Fokusgruppe Artificial Intelligence for Health der International Telecommunication Union/World Health Organization (ITU/WHO) können für zahnärztliche Versorgungsforschung sinnvolle Ansprechpartner sein.

## Chancen und Hürden – Call for Action

Eine Reihe von Chancen und Hürden für zahnärztliche Versorgungsforschung lässt sich identifizieren: Die zahnärztliche Versorgung in Deutschland ist im Europavergleich auf hohem Niveau, weist nichtsdestotrotz aber eine Reihe systemimmanenter Beschränkungen auf [40]. *Vergleichende Versorgungsforschung* dürfte geeignet sein, erfolgreiche Strukturen aus Deutschland gegen andere Versorgungsformen zu evaluieren und in andere Systeme zu transportieren; umgekehrt sollten innovative und funktionierende Ansätze aus anderen Versorgungsumgebungen auf ihre Passform für Deutschland überprüft und bei Bedarf adaptiert werden.

Der langfristige Ausbau einer *Professur an jedem Studienstandort der Zahnmedizin* bietet die Möglichkeit, dass Absolventen der für Versorgungswissenschaften einschlägigen Fächer (Volkswirtschaftslehre, Gesundheitswissenschaften, Public Health usw.) sich mit den Besonderheiten und Facetten der zahnärztlichen Versorgung vertraut machen und die Methodenkompetenz der zahnärztlichen Versorgungsforschung erhöhen. Dies ist auf dem Gebiet der Medizintechnik und dentalen Werkstoffwissenschaften beispielgebend gelungen.

Die *Einbindung von Lehrpraxen in der neuen AOZ* bietet die Chance einer engen Anbindung der niedergelassenen Zahnärzteschaft (Versorgungspraxis) an die Hochschulen in der Lehre wie auch der praxisbasierten Versorgungsforschung unter Einbeziehung der Zahnärztekammern, KZVen und wissenschaftlichen Fachgesellschaften.

Der mangelnde Zugang zu strukturiert erhobenen, robusten und repräsentativen Daten limitiert Forschungspotenziale. Datenschutzhürden und proprietäre, interessensgeleitete *Zugangsbeschränkungen sollten sinnvoll reduziert bzw. aufgehoben werden*. Von einem verantwortlichen Umgang und Erkenntnisgewinn aus medizinischen Daten profitieren möglicherweise Millionen Menschen.

Der Mangel an Strukturen und Ausstattung bleibt eine zentrale Hürde. Aus den o. g. derzeitigen Rahmenbedingungen (Lehrstühle, Ausbildung, Förderformate) kann nur bedingt eine breit vernetzte Versorgungsforschung geleistet werden; unsere Analyse der Aktivitäten zeigt jedoch ein *hohes Vernetzungspotenzial*. Auch sind derzeitige kapazitätsrechtlich begründete Organisationsformen von Forschung und Lehre (Abbildung der Lehrkapazität einer sinnfällig personell ausgestatteten Professur) nur bedingt geeignet, jegliche Form von Forschung und Nachwuchsentwicklung zu befördern.

## Fazit und Ausblick

Die zahnärztliche Versorgungsforschung in Deutschland zeigte in den letzten 10 Jahren – ohne eine breite strukturelle Etablierung und Förderung – eine beträchtliche Leistungsfähigkeit. Allerdings muss sie festen Anschluss an die Entwicklungen der Versorgungsforschung in Deutschland gewinnen und halten, um ein Versiegen des aufstrebenden „Pioniergeists“ zu verhindern. Hierzu sind nachhaltige (kapazitätsneutrale) Strukturen (Professuren, Mittelbau) und Rahmenbedingungen (Datennutzung, Förderung) notwendig, um den Ausbau von Methodenkompetenzen und themenbasierte Vernetzung (Schwerpunktstandorte) in der Zahnmedizin zu unterstützen. Grundlegend ist die Struktur der Forschungsförderung auf den Prüfstand zu stellen und die Gewichtung zwischen Grundlagenforschung und Versorgungsforschung neu zu bewerten. Forschungserkenntnisse sollten zielgerichtet und zeitnah evaluiert und in die Versorgung, Versorgungsplanung und -steuerung einfließen.

### Infobox Themenfelder für die zahnärztliche Versorgungsforschung bis 2040 [27, 28]

- Ungleiche und unfaire Verteilung von Erkrankungen des Mundes und der Zähne entlang von sozialen und ökonomischen Ungleichheiten und Risikofaktoren
- Den häufigsten Zahn- und Munderkrankungen liegen beeinflussbare Risikofaktoren (Zucker, Tabakkonsum, Alkohol, schlechte Hygiene) zugrunde; die Erkrankungen sind teilweise vermeidbar und teilen diese Risikofaktoren mit anderen chronischen Erkrankungen
- Mundgesundheit ist zentral für Wohlbefinden und Gesamtgesundheit. Ein universeller Zugang zur Zahnmedizin (jenseits von hohen und teils abschreckenden privaten Behandlungskosten) ist zwingend notwendig
- Der Fokus auf vertikale, erkrankungszentrierte Präventionsansätze reduziert die Wirksamkeit und Effizienz von Präventionsbemühungen. Ansätze jenseits klassischer zahnmedizinischer Settings (Praxen), also in Schulen oder am Arbeitsplatz, werden nur bedingt genutzt
- Der weltweite Mangel an Mundgesundheitsfachkräften und die Notwendigkeit einer Diversifizierung der Fachkräfte in vielen Gesundheitssystemen, um überhaupt einen oben beschriebenen universellen Zugang leisten zu können
- Der Mangel an aktualisierten, relevanten epidemiologischen Mundgesundheitsdaten. Die Notwendigkeit einer engmaschigen und integrierten Mundgesundheitsüberwachung, um politische Entscheidungen informiert treffen und etablierte Programme und Systeme evaluieren zu können. Insbesondere digitale Technologien sind hier vielversprechend
- Der Einfluss von Zahnmedizin auf den Planeten (Nachhaltigkeit zahnmedizinischer Versorgungen, Amalgam)
- Die Chancen einer digitalisierten Zahnmedizin (u. a. Telezahnmedizin, Datenzahnmedizin)

## Footnotes

1 (Germany[tiab] OR Germany[Mesh]) AND („health services research“ OR „economic evaluation“ OR „efficacy“ OR „inequalities“ OR „profession“ OR „access to care“ OR „patient satisfaction“ OR „patient autonomy“ OR „patient reported outcome“ OR cooperation OR „population based“ OR „secondary data“ OR „access to care“) AND („dental care“ OR „dentistry“[tiab] OR „oral health“ OR „dentist“ OR „dental practitioner“ OR „dental therapy“ OR „dental service“ OR „dental health“ OR periodontitis OR „oral implant“ OR „dental implant“ OR denture OR orthodontic OR „TMD“ OR „orofacial pain“) NOT (RCT OR „clinical trial“); bereinigt um Duplikate, Leitlinien und verbleibende klinische Studien. Daten verfügbar als 10.5281/zenodo.4756814.
